# Childhood sexual abuse and dissociation: Testing the betrayal trauma theory in two Chinese samples

**DOI:** 10.1192/j.eurpsy.2025.1476

**Published:** 2025-08-26

**Authors:** C. H. O. Huang, A. K. C. Chau, C. T. Y. Cheung, W. T. Chien, S. K. K. Lam, J. Y.-H. Wong, H. W. Fung

**Affiliations:** 1 Department of Social Work and Social Administration; 2 Department of Psychiatry, the University of Hong Kong, Pokfulam; 3 Department of Social Work, Hong Kong Baptist University, Kowloon; 4 The Nethersole School of Nursing, The Chinese University of Hong Kong, Shatin; 5 School of Nursing and Health Sciences, Hong Kong Metropolitan University; 6 School of Nursing, The Hong Kong Polytechnic University, Kowloon, Hong Kong

## Abstract

**Introduction:**

Childhood sexual abuse (CSA) is a major public health concern and is closely associated with dissociative symptoms. According to the betrayal trauma theory, dissociation can be interpreted as a response towards betrayal trauma (i.e., trauma perpetrated by a close person, such as a family member). No previous study has validated this theory with a focus on CSA in the Chinese context.

**Objectives:**

We hypothesized that people with betrayal CSA, but not non-betrayal CSA, would report significantly more dissociative symptoms than people without CSA. We also hypothesized that betrayal CSA, but not non-betrayal CSA, would be significantly associated with the severity of dissociative symptoms. We tested the hypotheses in two independent Chinese-speaking samples.

**Methods:**

Sample 1 (*N* = 91) consisted of participants seeking treatment in an evaluation study; while Sample 2 (*N* = 376) included community health service users in a survey study. In both Chinese-speaking samples, participants completed the two CSA items on the Brief Betrayal Trauma Survey and the 16-item Dissociative Features Section of the Self-report Dissociative Disorders Interview Schedule (SR-DDIS). ANOVA and regression analyses were used to test our hypotheses.

**Results:**

Across both samples, participants with betrayal CSA reported significantly more dissociative symptoms than those without any CSA (Sample 1: M = 5.60, SD = 3.14 vs M = 3.67, SD = 3.06; F = 3.301, p = .041, Sample 2: M = 2.06, SD = 2.49 vs M = 0.93, SD = 1.32; F = 8.428, p < .001). As hypothesized, no significant differences in dissociative symptoms were observed between participants with and without non-betrayal CSA (Sample 1: M = 4.00, SD = 3.71 vs M = 3.67, SD = 3.06; Sample 2: M = 1.14, SD = 1.83 vs M = .93, SD = 1.32). Across both samples, betrayal CSA, but not non-betrayal CSA, was significantly associated with dissociative symptoms (Sample 1: β = .250, p = .024, Sample 2: β = .189, p < .001), after controlling for age and gender.

**Conclusions:**

This study provides cross-cultural evidence for the betrayal trauma theory. We suggest that proactive family-centered child protection is needed to prevent CSA, and screening for dissociative symptoms is also necessary in CSA survivors.

**Disclosure of Interest:**

None Declared

